# *catena*-Poly[[{*N*,*N*-dimethyl-*N*′-[1-(pyridin-2-yl)ethyl­idene]ethane-1,2-diamine-κ^3^ *N*,*N*′,*N*′′}(thio­cyanato-κ*N*)cadmium]-μ-thio­cyanato-κ^2^ *S*:*N*]

**DOI:** 10.1107/S1600536811010063

**Published:** 2011-03-26

**Authors:** Nura Suleiman Gwaram, Hamid Khaledi, Hapipah Mohd Ali

**Affiliations:** a Department of Chemistry, University of Malaya, 50603 Kuala Lumpur, Malaysia

## Abstract

In the title compound, [Cd(NCS)_2_(C_11_H_17_N_3_)]_*n*_, the Cd^II^ atom is octa­hedrally coordinated by the *N*,*N*′,*N*′′-tridentate Schiff base ligand and one terminal thio­cyanate N atom. Two *trans*-*N:S*-bridging thio­cyanates complete the N_5_S donor set around the Cd atom. In the crystal, adjacent Cd^II^ ions are linked by the thio­cyanate *N:S*-bridges into polymeric chains along the *c* axis.

## Related literature

For the structures of some cadmium thio­cyanate complexes with nitro­gen-based ligands, see: Banerjee *et al.* (2005 ▶). For a singly bridged cadmium thio­cyanate complex, see: Bose *et al.* (2004 ▶). For a triply bridged cadmium thio­cyanate complex, see: Chen *et al.* (2002 ▶). For an S-bound terminal thio­cyanate cadmium complex, see: Nfor *et al.* (2006 ▶).
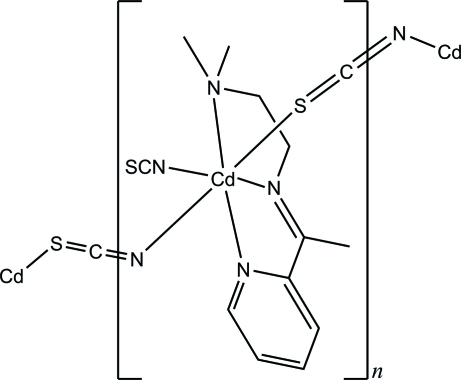


## Experimental

### 

#### Crystal data

[Cd(NCS)_2_(C_11_H_17_N_3_)]
*M* *_r_* = 419.84
Monoclinic, 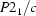

*a* = 14.602 (2) Å
*b* = 9.5827 (14) Å
*c* = 12.8714 (19) Å
β = 107.483 (2)°
*V* = 1717.9 (4) Å^3^
*Z* = 4
Mo *K*α radiation
μ = 1.51 mm^−1^
*T* = 100 K
0.35 × 0.29 × 0.08 mm

#### Data collection

Bruker APEXII CCD diffractometer
Absorption correction: multi-scan (*SADABS*; Sheldrick, 1996 ▶) *T*_min_ = 0.619, *T*_max_ = 0.889
19975 measured reflections
3756 independent reflections
3298 reflections with *I* > 2σ(*I*)
*R*_int_ = 0.047

#### Refinement

*R*[*F* ^2^ > 2σ(*F* ^2^)] = 0.023
*wR*(*F* ^2^) = 0.056
*S* = 1.07
3756 reflections
193 parameters
H-atom parameters constrained
Δρ_max_ = 0.54 e Å^−3^
Δρ_min_ = −0.73 e Å^−3^

### 

Data collection: *APEX2* (Bruker, 2007 ▶); cell refinement: *SAINT* (Bruker, 2007 ▶); data reduction: *SAINT*; program(s) used to solve structure: *SHELXS97* (Sheldrick, 2008 ▶); program(s) used to refine structure: *SHELXL97* (Sheldrick, 2008 ▶); molecular graphics: *X-SEED* (Barbour, 2001 ▶); software used to prepare material for publication: *SHELXL97* and *publCIF* (Westrip, 2010 ▶).

## Supplementary Material

Crystal structure: contains datablocks I, global. DOI: 10.1107/S1600536811010063/om2412sup1.cif

Structure factors: contains datablocks I. DOI: 10.1107/S1600536811010063/om2412Isup2.hkl

Additional supplementary materials:  crystallographic information; 3D view; checkCIF report

## Figures and Tables

**Table 1:** Selected bond lengths (Å)

Cd1—N4	2.2406 (18)
Cd1—N5	2.3008 (19)
Cd1—N2	2.3345 (17)
Cd1—N1	2.3801 (18)
Cd1—N3	2.3820 (19)
Cd1—S2	2.7803 (6)

Symmetry code: (i) 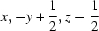.

**Fig. 1.**
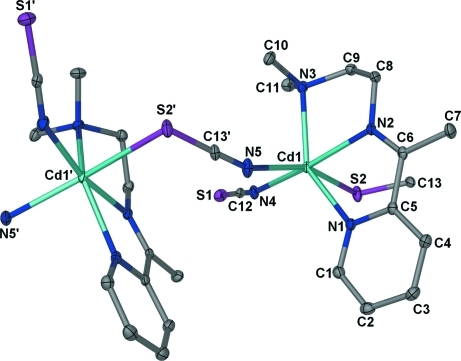
Thermal ellipsoid plot of the title compound at the 30% probability level. Hydrogen atoms have been omitted for clarity. Symmetry code: ' = x, –y+1/2, z - 1/2.

## supplementary crystallographic information

### Comment

Thiocyanate anion is known to bind the cadmium ion in different modes: terminal *N*-bound, terminal *S*-bound (Nfor *et al.* 2006) or *N:S*-bridging ligand. As a bridging ligand, it may give rise to a singly bridged (Bose *et al.* 2004), doubly bridged or triply bridged (Chen *et al.* 2002) cadmium complex. The title compound is a mixed-ligand cadmium complex with thiocyanate and the Schiff base *N,N*-dimethyl-*N'*-[methyl(2-pyridyl)methylene]ethane-1,2-diamine. Similar to what was observed in the cadmium thiocyanate adduct of the similar Schiff base, *N,N*-diethyl-*N'*-[methyl(2-pyridyl)methylene]ethane-1,2-diamine (Banerjee *et al.* 2005), the thiocyanate ions act as either bridging or terminal ligands. However, different from the doubly bridged dimeric structure of the former, in the present structure the bridging thiocyanate ligands singly bridge the adjacent metal centers, related by symmetry *x*, –*y+*1/2, *z* - 1/2, into infinite chains along the *c* axis. Within this coordination polymer, the Cd^II^ ions are separated by the distance of 8.0234 (9) Å. Two thiocyanate *N:S*-bridges, one terminal thiocyanate N atom and the *N,N',N"*-tridentate Schiff base make a distorted octahedral geometry around the Cd(II) atoms.

### Experimental

A mixture of 2-acetylpyridine (0.2 g, 1.65 mmol) and *N,N*-dimethylethyldiamine (0.15 g, 1.65 mmol) in ethanol (20 ml) was refluxed for 2 hr followed by addition of a solution of cadmium(II) acetate dihydrate (0.44 g, 1.65 mmol) and sodium thiocyanate (0.27 g, 3.3 mmol) in a minimum amount of water. The resulting solution was refluxed for 30 min, then set aside at room temperature. The crystals of the title compound were obtained in a few days.

### Refinement

Hydrogen atoms were placed at calculated positions at distances C—H = 0.95, 0.98 and 0.99 Å for aryl, methyl and methylene type H-atoms, respectively, and were treated as riding on their parent atoms, with U*iso*(H) = 1.2–1.5 times U*eq*(C).

### Crystal data

**Table:**

[Cd(NCS)_2_(C_11_H_17_N_3_)]	*F*(000) = 840
*M**_r_* = 419.84	*D*_x_ = 1.623 Mg m^−^^3^
Monoclinic, *P*2_1_/*c*	Mo *K*α radiation, λ = 0.71073 Å
Hall symbol: -P 2ybc	Cell parameters from 9975 reflections
*a* = 14.602 (2) Å	θ = 2.6–31.2°
*b* = 9.5827 (14) Å	µ = 1.51 mm^−^^1^
*c* = 12.8714 (19) Å	*T* = 100 K
β = 107.483 (2)°	Needle, colorless
*V* = 1717.9 (4) Å^3^	0.35 × 0.29 × 0.08 mm
*Z* = 4	

### Data collection

**Table:**

Bruker APEXII CCD diffractometer	3756 independent reflections
Radiation source: fine-focus sealed tube	3298 reflections with *I* > 2σ(*I*)
graphite	*R*_int_ = 0.047
φ and ω scans	θ_max_ = 27.0°, θ_min_ = 2.7°
Absorption correction: multi-scan (*SADABS*; Sheldrick, 1996)	*h* = −18→18
*T*_min_ = 0.619, *T*_max_ = 0.889	*k* = −12→12
19975 measured reflections	*l* = −16→16

### Refinement

**Table:**

Refinement on *F*^2^	Primary atom site location: structure-invariant direct methods
Least-squares matrix: full	Secondary atom site location: difference Fourier map
*R*[*F*^2^ > 2σ(*F*^2^)] = 0.023	Hydrogen site location: inferred from neighbouring sites
*wR*(*F*^2^) = 0.056	H-atom parameters constrained
*S* = 1.07	*w* = 1/[σ^2^(*F*_o_^2^) + (0.0162*P*)^2^ + 1.0805*P*] where *P* = (*F*_o_^2^ + 2*F*_c_^2^)/3
3756 reflections	(Δ/σ)_max_ = 0.001
193 parameters	Δρ_max_ = 0.54 e Å^−^^3^
0 restraints	Δρ_min_ = −0.72 e Å^−^^3^

### Special details

**Table:**

Geometry. All e.s.d.'s (except the e.s.d. in the dihedral angle between two l.s. planes) are estimated using the full covariance matrix. The cell e.s.d.'s are taken into account individually in the estimation of e.s.d.'s in distances, angles and torsion angles; correlations between e.s.d.'s in cell parameters are only used when they are defined by crystal symmetry. An approximate (isotropic) treatment of cell e.s.d.'s is used for estimating e.s.d.'s involving l.s. planes.
Refinement. Refinement of *F*^2^ against ALL reflections. The weighted *R*-factor *wR* and goodness of fit *S* are based on *F*^2^, conventional *R*-factors *R* are based on *F*, with *F* set to zero for negative *F*^2^. The threshold expression of *F*^2^ > σ(*F*^2^) is used only for calculating *R*-factors(gt) *etc*. and is not relevant to the choice of reflections for refinement. *R*-factors based on *F*^2^ are statistically about twice as large as those based on *F*, and *R*- factors based on ALL data will be even larger.

### Fractional atomic coordinates and isotropic or equivalent isotropic displacement parameters (Å^2^)

**Table:**

	*x*	*y*	*z*	*U*_iso_*/*U*_eq_	
Cd1	0.234091 (10)	0.221072 (15)	0.070761 (11)	0.01927 (6)	
S1	0.37258 (4)	−0.22496 (6)	0.01149 (6)	0.03326 (14)	
S2	0.23783 (5)	0.02652 (6)	0.23222 (5)	0.03374 (15)	
N1	0.07134 (13)	0.27035 (18)	0.05811 (14)	0.0217 (4)	
N2	0.22324 (12)	0.41643 (18)	0.17479 (13)	0.0208 (4)	
N3	0.39458 (13)	0.30157 (18)	0.14777 (15)	0.0234 (4)	
N4	0.26943 (14)	0.0221 (2)	−0.00030 (15)	0.0279 (4)	
N5	0.20178 (16)	0.3205 (2)	−0.09914 (16)	0.0353 (5)	
C1	−0.00422 (17)	0.2001 (2)	−0.00390 (18)	0.0265 (5)	
H1	0.0067	0.1264	−0.0481	0.032*	
C2	−0.09807 (18)	0.2296 (2)	−0.0070 (2)	0.0307 (5)	
H2	−0.1502	0.1779	−0.0527	0.037*	
C3	−0.11374 (16)	0.3359 (3)	0.05798 (19)	0.0298 (5)	
H3	−0.1770	0.3575	0.0589	0.036*	
C4	−0.03583 (16)	0.4107 (2)	0.12192 (18)	0.0271 (5)	
H4	−0.0452	0.4844	0.1671	0.033*	
C5	0.05605 (15)	0.3770 (2)	0.11946 (16)	0.0210 (4)	
C6	0.14267 (15)	0.4573 (2)	0.18289 (16)	0.0212 (4)	
C7	0.12885 (17)	0.5799 (2)	0.25002 (18)	0.0288 (5)	
H7A	0.1180	0.5461	0.3172	0.043*	
H7B	0.0732	0.6345	0.2082	0.043*	
H7C	0.1864	0.6388	0.2683	0.043*	
C8	0.31469 (16)	0.4832 (2)	0.23056 (18)	0.0255 (5)	
H8A	0.3129	0.5220	0.3012	0.031*	
H8B	0.3272	0.5605	0.1857	0.031*	
C9	0.39357 (16)	0.3735 (2)	0.24905 (17)	0.0257 (5)	
H9A	0.4566	0.4190	0.2817	0.031*	
H9B	0.3846	0.3035	0.3017	0.031*	
C10	0.42503 (18)	0.3962 (2)	0.0739 (2)	0.0310 (5)	
H10A	0.4891	0.4329	0.1112	0.047*	
H10B	0.3794	0.4736	0.0527	0.047*	
H10C	0.4268	0.3447	0.0087	0.047*	
C11	0.46237 (17)	0.1834 (3)	0.1762 (2)	0.0332 (5)	
H11A	0.4639	0.1353	0.1096	0.050*	
H11B	0.4415	0.1181	0.2232	0.050*	
H11C	0.5267	0.2182	0.2148	0.050*	
C12	0.31368 (15)	−0.0797 (2)	0.00673 (16)	0.0226 (4)	
C13	0.21670 (16)	0.1178 (2)	0.33052 (17)	0.0260 (5)	

### Atomic displacement parameters (Å^2^)

**Table:**

	*U*^11^	*U*^22^	*U*^33^	*U*^12^	*U*^13^	*U*^23^
Cd1	0.02395 (9)	0.01941 (9)	0.01697 (9)	0.00128 (6)	0.01000 (6)	−0.00108 (5)
S1	0.0287 (3)	0.0286 (3)	0.0440 (4)	0.0061 (2)	0.0131 (3)	0.0010 (3)
S2	0.0595 (4)	0.0236 (3)	0.0247 (3)	0.0007 (3)	0.0226 (3)	0.0017 (2)
N1	0.0258 (9)	0.0225 (9)	0.0191 (9)	−0.0010 (7)	0.0103 (7)	0.0004 (7)
N2	0.0246 (9)	0.0216 (9)	0.0181 (8)	0.0009 (7)	0.0092 (7)	−0.0013 (7)
N3	0.0233 (9)	0.0225 (9)	0.0267 (10)	0.0032 (7)	0.0110 (8)	0.0003 (7)
N4	0.0385 (11)	0.0240 (10)	0.0254 (10)	0.0025 (8)	0.0160 (8)	−0.0052 (8)
N5	0.0485 (13)	0.0398 (12)	0.0220 (10)	0.0145 (10)	0.0175 (9)	0.0068 (9)
C1	0.0308 (12)	0.0250 (12)	0.0246 (11)	−0.0028 (9)	0.0096 (9)	−0.0011 (9)
C2	0.0286 (12)	0.0326 (13)	0.0298 (12)	−0.0068 (10)	0.0071 (10)	0.0041 (10)
C3	0.0244 (11)	0.0348 (13)	0.0327 (12)	0.0014 (10)	0.0120 (10)	0.0065 (10)
C4	0.0271 (12)	0.0297 (12)	0.0273 (11)	0.0035 (9)	0.0123 (9)	0.0021 (9)
C5	0.0262 (11)	0.0226 (10)	0.0163 (9)	0.0013 (8)	0.0094 (8)	0.0035 (8)
C6	0.0287 (11)	0.0216 (10)	0.0145 (9)	0.0033 (8)	0.0084 (8)	0.0030 (8)
C7	0.0305 (12)	0.0306 (12)	0.0253 (11)	0.0066 (10)	0.0084 (9)	−0.0062 (9)
C8	0.0270 (11)	0.0263 (11)	0.0252 (11)	−0.0034 (9)	0.0109 (9)	−0.0066 (9)
C9	0.0240 (11)	0.0285 (12)	0.0239 (11)	−0.0020 (9)	0.0061 (9)	−0.0008 (9)
C10	0.0355 (13)	0.0274 (12)	0.0374 (13)	−0.0006 (10)	0.0220 (11)	0.0000 (10)
C11	0.0263 (12)	0.0304 (12)	0.0412 (14)	0.0079 (10)	0.0078 (10)	0.0026 (11)
C12	0.0239 (11)	0.0280 (11)	0.0182 (10)	−0.0064 (9)	0.0097 (8)	−0.0032 (8)
C13	0.0318 (12)	0.0282 (11)	0.0187 (10)	−0.0093 (9)	0.0087 (9)	0.0006 (9)

### Geometric parameters (Å, °)

**Table:**

Cd1—N4	2.2406 (18)	C3—C4	1.387 (3)
Cd1—N5	2.3008 (19)	C3—H3	0.9500
Cd1—N2	2.3345 (17)	C4—C5	1.390 (3)
Cd1—N1	2.3801 (18)	C4—H4	0.9500
Cd1—N3	2.3820 (19)	C5—C6	1.496 (3)
Cd1—S2	2.7803 (6)	C6—C7	1.507 (3)
S1—C12	1.628 (2)	C7—H7A	0.9800
S2—C13	1.642 (2)	C7—H7B	0.9800
N1—C1	1.333 (3)	C7—H7C	0.9800
N1—C5	1.351 (3)	C8—C9	1.524 (3)
N2—C6	1.274 (3)	C8—H8A	0.9900
N2—C8	1.460 (3)	C8—H8B	0.9900
N3—C10	1.475 (3)	C9—H9A	0.9900
N3—C11	1.476 (3)	C9—H9B	0.9900
N3—C9	1.479 (3)	C10—H10A	0.9800
N4—C12	1.159 (3)	C10—H10B	0.9800
N5—C13^i^	1.155 (3)	C10—H10C	0.9800
C1—C2	1.388 (3)	C11—H11A	0.9800
C1—H1	0.9500	C11—H11B	0.9800
C2—C3	1.380 (3)	C11—H11C	0.9800
C2—H2	0.9500	C13—N5^ii^	1.155 (3)
			
N4—Cd1—N5	88.34 (7)	C3—C4—H4	120.3
N4—Cd1—N2	168.52 (7)	C5—C4—H4	120.3
N5—Cd1—N2	100.55 (7)	N1—C5—C4	121.3 (2)
N4—Cd1—N1	119.25 (7)	N1—C5—C6	116.56 (18)
N5—Cd1—N1	86.41 (7)	C4—C5—C6	122.14 (19)
N2—Cd1—N1	69.01 (6)	N2—C6—C5	116.59 (18)
N4—Cd1—N3	97.29 (7)	N2—C6—C7	124.9 (2)
N5—Cd1—N3	99.00 (7)	C5—C6—C7	118.48 (18)
N2—Cd1—N3	74.30 (6)	C6—C7—H7A	109.5
N1—Cd1—N3	143.28 (6)	C6—C7—H7B	109.5
N4—Cd1—S2	77.26 (5)	H7A—C7—H7B	109.5
N5—Cd1—S2	160.16 (6)	C6—C7—H7C	109.5
N2—Cd1—S2	95.65 (4)	H7A—C7—H7C	109.5
N1—Cd1—S2	88.81 (4)	H7B—C7—H7C	109.5
N3—Cd1—S2	96.33 (5)	N2—C8—C9	108.16 (17)
C13—S2—Cd1	104.63 (8)	N2—C8—H8A	110.1
C1—N1—C5	118.65 (19)	C9—C8—H8A	110.1
C1—N1—Cd1	124.79 (15)	N2—C8—H8B	110.1
C5—N1—Cd1	116.56 (14)	C9—C8—H8B	110.1
C6—N2—C8	123.78 (18)	H8A—C8—H8B	108.4
C6—N2—Cd1	121.13 (14)	N3—C9—C8	113.02 (18)
C8—N2—Cd1	115.06 (12)	N3—C9—H9A	109.0
C10—N3—C11	108.77 (18)	C8—C9—H9A	109.0
C10—N3—C9	111.49 (17)	N3—C9—H9B	109.0
C11—N3—C9	108.82 (18)	C8—C9—H9B	109.0
C10—N3—Cd1	112.34 (14)	H9A—C9—H9B	107.8
C11—N3—Cd1	110.94 (14)	N3—C10—H10A	109.5
C9—N3—Cd1	104.40 (12)	N3—C10—H10B	109.5
C12—N4—Cd1	151.16 (18)	H10A—C10—H10B	109.5
C13^i^—N5—Cd1	157.5 (2)	N3—C10—H10C	109.5
N1—C1—C2	123.1 (2)	H10A—C10—H10C	109.5
N1—C1—H1	118.4	H10B—C10—H10C	109.5
C2—C1—H1	118.4	N3—C11—H11A	109.5
C3—C2—C1	118.4 (2)	N3—C11—H11B	109.5
C3—C2—H2	120.8	H11A—C11—H11B	109.5
C1—C2—H2	120.8	N3—C11—H11C	109.5
C2—C3—C4	119.0 (2)	H11A—C11—H11C	109.5
C2—C3—H3	120.5	H11B—C11—H11C	109.5
C4—C3—H3	120.5	N4—C12—S1	177.5 (2)
C3—C4—C5	119.5 (2)	N5^ii^—C13—S2	178.5 (2)

Symmetry codes: (i) *x*, −*y*+1/2, *z*−1/2; (ii) *x*, −*y*+1/2, *z*+1/2.
